# Electrical Detection of C-Reactive Protein Using a Single Free-Standing, Thermally Controlled Piezoresistive Microcantilever for Highly Reproducible and Accurate Measurements

**DOI:** 10.3390/s130809653

**Published:** 2013-07-29

**Authors:** Yi-Kuang Yen, Yu-Cheng Lai, Wei-Ting Hong, Yotsapoom Pheanpanitporn, Chuin-Shan Chen, Long-Sun Huang

**Affiliations:** 1 Institute of Applied Mechanics, National Taiwan University, Taipei 10617, Taiwan; E-Mails: ykyen@ntu.edu.tw (Y.-K.Y.); yclai@ntumems.net (Y.-C.L.); wthong@ntumems.net (W.-T.H.); ypchang@ntumems.net (Y.P.); 2 Department of Civil Engineering, National Taiwan University, Taipei 10617, Taiwan; E-Mail: dchen@ntu.edu.tw

**Keywords:** C-reactive protein, biosensor, piezoresistive microcantilever, reproducibility, MEMS

## Abstract

This study demonstrates a novel method for electrical detection of C-reactive protein (CRP) as a means of identifying an infection in the body, or as a cardiovascular disease risk assay. The method uses a single free-standing, thermally controlled piezoresistive microcantilever biosensor. In a commonly used sensing arrangement of conventional dual cantilevers in the Wheatstone bridge circuit, reference and gold-coated sensing cantilevers that inherently have heterogeneous surface materials and different multilayer structures may yield independent responses to the liquid environmental changes of chemical substances, flow field and temperature, leading to unwanted signal disturbance for biosensing targets. In this study, the single free-standing microcantilever for biosensing applications is employed to resolve the dual-beam problem of individual responses in chemical solutions and, in a thermally controlled system, to maintain its sensor performance due to the sensitive temperature effect. With this type of single temperature-controlled microcantilever sensor, the electrical detection of various CRP concentrations from 1 μg/mL to 200 μg/mL was performed, which covers the clinically relevant range. Induced surface stresses were measured at between 0.25 N/m and 3.4 N/m with high reproducibility. Moreover, the binding affinity (K_D_) of CRP and anti-CRP interaction was found to be 18.83 ± 2.99 μg/mL, which agreed with results in previous reported studies. This biosensing technique thus proves valuable in detecting inflammation, and in cardiovascular disease risk assays.

## 1. Introduction

Microcantilever-based sensors are a promising tool for biological studies [1], clinical diagnosis [2,3], environmental monitoring [4,5], functional genomics [6,7] and pharmacological drug screening [8]. Due to the fact that molecular recognition induces surface stress or deflection on a microcantilever [9,10], a growing number of studies have reported that microcantilever sensors showed excellent detection of antibody-antigen binding [2,3], DNA hybridization [6,7], and enzyme reactions [11], and also performed well in drug screenings [8]. Although fluorescence-based detection is still the most popular approach [12], the technique is complicated, involving labeling the target biomolecules with a dye, expensive arrays and equipment for fluorescence analysis. Light-weight, with no need for fluorescence labeling, and highly compatible with integrated circuits, the microcantilever label-free biosensing technique provides high sensitivity and miniaturization, and is therefore potentially portable as part of a point-of-care platform or personal diagnosis apparatus. For point-of-care or tele-health applications, real-time investigation and miniaturization of an instrument are required. For most clinical applications, disease-related protein biomarkers appear to be appropriate target candidates, because proteins are the most abundant biological macromolecules found in human blood, and are also the final product to show the information pathways of biological signals. Several studies have demonstrated disease-related biomarkers for prostate cancer [2,3], cardiovascular disease [13,14] and Hepatocellular carcinoma [15]. This study focuses on C-reactive protein (CRP), which is a classical acute phase protein produced in the liver, and an important biomarker in acute infection or inflammation. CRP is usually present in healthy human serum at a concentration of less than 1 μg/mL. CRP's molecular weight is around 115 kDa, and it consists of five identical subunits which form a circular structure. CRP levels can be elevated as much as 100 or even 500 times during acute infection or inflammation anywhere in the body. Recently, numerous epidemiological studies have shown that CRP is associated with an important risk factor used to assess cardiovascular disease [16,17], and has often been used as an indicator in health examinations.

In order to detect CRP, the measurement of microcantilever deflection can be performed at the optical level, or with the electrical transduction technique. The optical level technique reflects a laser off the end of a cantilever onto a detector, and is able to amplify the minute cantilever deflections. This technique is widely used in atomic force microscopy. Alternatively, an electrical transducer can be used to convert a microcantilever deflection into an electrical signal. This system can be miniaturized so as to be potentially portable for point-of-care or personal diagnosis [18,19]. In addition to these methods, two free-standing piezoresistive microcantilevers of reference and sensing structures integrated with Wheatstone bridge electrical circuitry are commonly used for thermally insensitive configuration [3,20], while another method using electrical transduction by a field-effect transistor based on a silicon-on-insulator (SOI) substrate [21,22] is still under development, although it is expected to be potentially costly. In biosensing applications, the conventional dual-beam, free-standing microcantilever configuration has been extensively used for the detection of chemical interaction and biomolecular recognition in liquid environments. For that purpose, an additional layer of gold on a sensing beam is required for sensing surface treatment, which might induce minute differences of surface curvature and thermal properties from the reference beam. Moreover, piezoresistive microcantilevers that incorporate bio-recognition elements as receptors so as to interact with specific molecules are able to convert molecular recognition into an electrical signal. The recognition element-coated microcantilever exhibits a certain degree of sensitivity to pH, ionic, or chemical liquid environments [23,24]. As a result of the heterogeneous surface material properties for both free-standing sensing and reference microcantilevers, the detection of chemical interaction and biomolecular recognition in conventional dual-beam free-standing configuration may lead to unexpected or irreproducible results in a biochemical environment.

In this study, a single free-standing piezoresistive microcantilever for electrical detection is employed in order to improve on the independent responses generated by conventional dual-beam free-standing microcantilevers for chemical interaction or bio-molecular recognition. Single free-standing piezoresistive microcantilevers and microchannels are micromachined, packaged, and assembled in a small form factor. Measurement of the microfluidic microcantilever biosensor is conducted in a thermally controlled environment at 0.1 °C. Measurement of CRP concentrations and calculation of its binding affinity are performed in order to improve the precision and reproducibility of the single free-standing microcantilever biosensor.

## 2. Experimental Section

**Figure 1.**
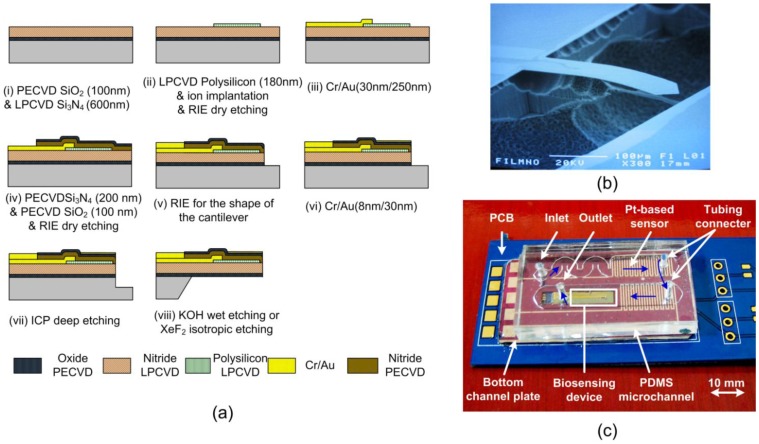
(**a**) Fabrication process of a single free-standing, thermally steady piezoresistive microcantilever for biosensing applications; (**b**) The SEM picture of the single free-standing piezoresistive microcantilever with no reference microcantilever; (**c**) Photograph of the electrically detected sensor-inside packaged micro channel device.

As shown in Figure 1, the overall microfluidic biosensing packaged device consists of a piezoresistive microcantilever sensor chip, a microchannel with a micromachined silicon bottom pedestal and a PDMS-based microchannel capping structure, and a PCB with essential electronic components. The packaged device provides the sensor with a small stable liquid chamber for biological and chemical reactions.

### 2.1. Design of Single Free-Standing Piezoresistive Microcantilever

The piezoresistive microcantilever is composed of several layers, including: a structural insulation layer of nitride (Si_x_N_y_) and oxide, a piezoresistive polysilicon layer, an insulation layer of nitride, a gold layer for surface reactions, and a passivation layer of nitride. The thickness, Young's modulus, residual stress and process of each thin film layer determine the resultant built-in stress of the composite structure. If the built-in stress between the layers of a microcantilever is not carefully designed, it may result in the microcantilever bending after a releasing process, which may increase the noise signal due to the mechanical disturbance caused by a liquid flow [25].

First, the silicon nitride layer is used as a structural layer, which is the main material of the microcantilever structure. Moreover, it also plays important roles as the passivation layer, which protects the electrically piezoresistive layer from any liquids during the fabrication processes, and as the insulation layer, which ensures that the electrical sensor can work in a liquid environment. Secondly, the electrical property of the polysilicon layer strongly depends on the doping level and annealing conditions, which also reduce a certain degree of residual stress. The thin films of silicon oxide are positioned on the top and bottom sides of the microcantilever in order to balance the built-in stress.

The resultant built-in stresses after release tend to be a linear variation of stresses forming a maximum magnitude at the top surface and a minimum value on the opposite bottom surface [26]. In a composite structure, the neutral axis of a microcantilever at an unstressed level in direction of thickness is considered to be the appropriate position to place piezoresistive layer. Meanwhile, the distance between the neutral axis and the piezoresistor layer also affects the surface stress sensitivity. As a result, it is suggested that the piezoresistor layer be kept as thin as possible, and that it be placed as far as possible from the neutral axis in order to gain the maximum induced surface stress. The neutral axis Z_N_ is defined as:

(1) $$Z_{N}=\frac{\sum_{i}E_{i}z_{i}t_{i}}{\sum_{i}E_{i}t_{i}}$$

where *E_i_* is the Young's modulus of the i'th-layer, *z_i_* is the distance of the i'th-layer from the neutral axis and *t_i_* is the thickness of the i'th-layer. From these parameters, the neutral axis Z_N_ is calculated to be 585.65 nm in this study, which is located at the structural silicon nitride layer. The chosen dimensions for the microcantilevers in the final design are; length 200 μm, width 60 μm, and thickness is about 1.2 μm. The length of the piezoresistor is 120 μm, and it is 180 nm thick.

The surface stress sensitivity of a piezoresistive microcantilever is defined by the piezoresistive voltage over the surface stress from a biochemical reaction. The surface stress sensitivity is related to both two-dimensional strain and piezo-coefficient in the piezoresistive layer [27]. The surface stress sensitivity can be expressed as [26]:

(2) $$\frac{\Delta R}{R}=K\left(-\frac{1}{\sum_{i}E_{i}t_{i}}-\frac{z_{R}z_{T}}{\sum_{i}E_{i}t_{i}\left({\left(z_{T}-\sum_{j=0}^{i}t_{j}+\frac{t_{i}}{2}\right)}^{2}+\frac{1}{3}{\left(\frac{t_{i}}{2}\right)}^{2}\right)}\right)\sigma_{s}$$

where *R* and Δ*R* are the piezoresistor resistance and its relative change in resistance, *K* is the gauge factor, *Z_T_* is the distance from the top of the cantilever to the neutral axis, *Z_R_* is the distance between the piezoresistor and the neutral plane, and *σ_s_* is the surface stress. In this study, surface stress sensitivity can be designed and calculated to be 3.62 × 10^−4^ (N/m)^−1^ for a gold coated microcantilever. Meanwhile, an output voltage of 1 μV corresponds to a surface stress change of 32.5 mN/m.

### 2.2. Fabrication of Single Free-Standing Piezoresistive Microcantilever

The sensor chip was fabricated using the MEMS micromachining process. An illustration of the single free-standing piezoresistive microcantilever fabrication process is given in Figure 1a. A 100-nm-thick silicon oxide layer was first evaporated onto a silicon wafer by the plasma-enhanced chemical vapor deposition (PECVD) technique, and a 600-nm-thick low-stress silicon nitride was deposited as the structural layer by the low-pressure chemical-vapor deposition (LPCVD) technique. A 180-nm-thick polycrystalline silicon (poly-Si) layer was deposited on the silicon nitride layer as a piezoresistive material by the LPCVD technique, followed by the boron implantation at 30 keV with a dose of 5 × 10^15^ cm^−2^ and annealing at 1,050 °C for 30 min. Subsequently, the piezoresistors were patterned and defined using reactive ion etching. A 30/250-nm-thick Cr/Au layer was deposited using an e-beam evaporator, and then etched in solution to define the connection wires and bonding pads. After this, a 200-nm-thick PECVD silicon nitride layer and a 100-nm-thick PECVD silicon oxide layer were sequentially deposited as an insulation layer and as an etching mask for the later releasing process, respectively. The shape of microcantilever was defined using reactive ion etching. A 30-nm-thick gold layer was deposited as the sensing layer for the immobilization of chemical probes. In order to shorten the time required for wet etching, a deep-reactive ion etching (DRIE) was initially used to etch the silicon substrate. The finished piezoresistive microcantilever was then released by etching the silicon substrate in a KOH solution. The SEM image of the complete piezoresistive microcantilever is shown in Figure 1b.

### 2.3. Packaging and Assembly

First, the device provided a small volume reaction chamber, which decreased the amount of analytes needed. The flow in a microchannel can be considered a laminar flow at a suitable flow rate. The insulating materials inside the microchannel were required to be waterproof and unreactive with any chemical substances. The microchannel was designed to be 4,000 μm wide, and 240 μm deep. The flow rate in the channel was 10 μL/min. The Reynolds number of water flowing in the channel was calculated to be 0.09. Second, the channel plate served as the bottom pedestal of the microfluidic channel. In order to monitor the temperature variation surrounding the reaction zone in the channel, the platinum resistance temperature detector (RTD) operating as a temperature sensor was integrated on the channel base. Two platinum RTD temperature sensors were placed at the inlet and outlet of the microchannel to give feedback to the temperature-control system, as shown in Figure 1c. The temperature sensors were designed in a serpentine shape to increase the contact area with the flow. The RTD temperature sensors were fabricated by depositing platinum as a thin film, and detecting temperature changes as its resistance linearly varied.

The overall package consisted of a sensor chip, a microchannel, a channel plate and a PCB with essential electronic components. The piezoresistive microcantilever chip was first attached to the PCB, and then bonding pads on a sensor were wire bonded to the connecting pads on the PCB, and were insulated by gluing. Next, the channel bottom plate was adhered to the PCB and coated with a thin PDMS layer in order to insulate a circuit on the channel bottom plate. After this, the PDMS microchannel was adhered to the channel bottom plate. Finally, steel tubes were inserted into the PDMS microchannel as the inlet and outlet. The complete PCB-based piezoresistive microcantilever sensor package is shown in Figure 1c.

### 2.4. Experimental Setup of Detection System

The package contained a microchannel and an embedded temperature sensor on the PCB, which was connected to an electrical readout system. The package provided the sensor with a small, stable liquid chamber for biological and chemical reactions. In addition, a platinum temperature sensor was included for the real-time temperature monitoring for thermal effect investigation and compensation. The PCB-based package offered the advantages of low cost and ease of integration into other electrical systems. The setup of a signal acquisition system was designed for experimental and portable use. A chip consisting of a gold-coated microcantilever and a bare Si_3_N_4_ microcantilever were required to configure the Wheatstone bridge circuit for the signal readout. The aim of using dual microcantilevers was to find the different responses of two cantilevers to the variation of chemical concentrations in a liquid environment.

The electrical detection system showed the signal generated by the bending of the piezoresistive microcantilever, which was caused by the biochemical recognition reaction. Since bio-recognition signals were usually very small in a range of about 1–10 μV, the signal needed to be amplified 5,000-fold, and transmitted to the analog input region of the analog-to-digital converter (ADC). The detection system also needed to filter out low-frequency noise generated by the environment and the sensor itself.

The cryostat system consisted of a heat insulating box and a cryostat tank. The box was 12 cm in length, 8 cm in width and 5 cm high. The box was designed to be heat insulated from the outside environment. The cryostat tank was able to maintain temperatures ranging from 0 to 100 °C, with a temperature precision of ±0.1 °C. The piezoresistive microcantilever sensor was placed inside the box, with the platinum temperature sensor to monitor the temperature. Temperature variation was monitored for a significant time. Except for the drastic change at the beginning of the measurement, further temperature variations were within 0.1 °C.

### 2.5. Sample Preparation

The procedure for the immobilization of capture proteins on a microcantilever sensor chip is described in the following. The microcantilever chip was cleaned with HCI and NaOH sequentially and rinsed in deionized water. Then, 8-mercaptooctanoic acid (MOA, SH-(CH_2_)_7_-COOH, 20 mM) dissolved in 95% ethanol was injected into the device and incubated for 2 h. After the linker molecules were covalently chemisorbed on the gold coated surface of the microcantilever, this reactive self-assembled monolayer was activated by a mixed solution of EDC and NHS [18]. Separate vials containing 200 μL of 0.1 M N-hydroxysuccinimide (NHS), 0.4 M N-ethyl-N'-(3-dimethylaminopropyl)-carbodiimide hydrochloride (EDC), 1 M ethanolamine hydrochloride solution and a vial for mixing EDC and NHS were prepared. A continuous-flow pump was filled with distilled phosphate-buffered saline (PBS) at pH 7.2. Once the sensor device conditioned with PBS reached a steady state, the immobilization process was performed at a flow rate of 10 μL/min. 200-μL EDC and 200-μL NHS were each transferred to a mixing vial. A mixing solution of one hundred microliters was injected into the channel device. 100 μL of the capture protein (monoclonal anti-human CRP, mouse IgG1 isotype, Sigma C1688) was then injected, followed by 100 μL of ethanolamine hydrochloride solution. Finally, the analyte CRP (purity of ≥99%, Sigma C4063, St. Louis, MO, USA) was injected for detection.

## 3. Results and Discussion

### 3.1. Chemical Effect on Conventional Dual Free-Standing Microcantilevers

In this study, the chemical effect in a dual-beam arrangement arises in a chemical environment of selected ethanol or pH solution, which could lead to a false resulting signal. The electrical responses of both reference and sensing cantilevers were shown in this experiment to be independently reactive, and different in ethanol or pH tests with varied concentrations. If the dual beams are exposed to a complex body fluid environment of patient serum containing proteins, hormones, unknown drugs, antibodies, antigens *etc.*, the response of the reference cantilever could be unexpected. In order to avoid this uncertainty in the responses of reference cantilevers, a single free-standing sensing cantilever was proposed in this study to circumvent the unexpected chemical disturbances for biochemical measurement.

#### 3.1.1. Ethanol Test

**Figure 2.**
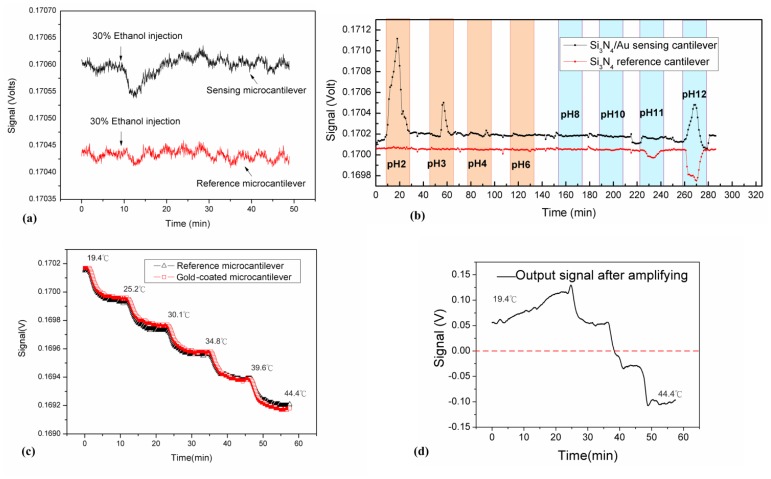
(**a**) The output signals of conventional dual microcantilevers as a function of time. At the 9th minute, 50 μL of 30% ethanol solution was injected. The response of the sensing cantilever to the ethanol immediately changed; (**b**) The bending responses of a Si_3_N_4_/Au sensing microcantilever and a Si_3_N_4_ reference microcantilever were measured in solutions as a function of time in a range from pH 2 to pH 12; (**c**) Voltages between two bridge terminals were measured, which indicated the resistivity change of piezoresistors on both the gold-coated and reference cantilevers as the temperature changed. The output voltage of the full-bridge showed the signal variation due to the temperature variation. Meanwhile, the responses shown in (a–c) were raw signals; (**d**) It was not possible to predict whether the resultant signal obtained by subtracting the reference cantilever response from that of the sensing one would be a positive or negative value. Meanwhile, the output shown in (d) was the amplified signal.

In this experiment, 50 μL of 30% ethanol solution was injected into the reaction microchannel at a flow rate of 10 μL/min. The complete device utilized a conventional arrangement of free-standing sensing and reference microcantilevers possessed heterogeneous microcantilever surfaces of gold and silicon nitride in this study. In Figure 2a, the output voltage responses of the gold-coated sensing and bare Si_3_N_4_ reference microcantilevers are shown as a function of time. The output signals changed distinctly in the gold-coated sensing cantilever as the 30% ethanol solution arrived. As the ethanol solution passed through the channel, the signal returned to the base line. It was found that the changes in the output signal were about 80 μV and 40 μV for the sensing and reference microcantilevers, respectively.

The response of the sensing microcantilever was greater than that of the reference cantilever. The apparent change in response of the gold-coated sensing microcantilever may be due to the heterogeneous surface properties of the top and bottom sides of the microcantilever in the presence of the ethanol solution [20]. The signal returned to its base line as the 30% ethanol solution left the reaction chamber. Therefore, the result of both sensing and reference microcantilevers exposed to the ethanol solution exhibited independent responses.

#### 3.1.2. Test of Various pH Solutions

The experiment with dual free-standing microcantilevers in ionic solution was conducted by change of pH solutions [24,28]. As the surfaces of both free-standing microcantilevers exposed to the solution proportional to ionic strength accumulate charges, the bending responses measured from both Si_3_N_4_/Au and Si_3_N_4_ surfaces of microcantilevers are shown in Figure 2b. An output voltage change was observed when introducing solutions between pH 2 and pH 12 of ionic strength into the reaction chamber. Since the Si_3_N_4_ surface of the cantilever may interact with H+ ions, the greater deflection signals occurred in extremely low and high pH solutions [24].

The reference microcantilever was initially designed to eliminate or reduce environmental interferences, such as thermal disturbances. In chemical tests, the reference microcantilever showed the opposite response to that of the sensing one in a high pH solution. The presence of metallic and chemical coatings on the top surface of the microcantilever greatly affect the deflections of the gold-coated and the bare silicon nitride cantilevers when exposed to various pH solutions [24]. As a result, the bare Si_3_N_4_ reference microcantilever may interact with H^+^ ions reacting with the pH solution, which was expected to be inert in its exposed environment [6]. The unwanted response of the reference microcantilever due to chemical interactions in solution may influence the signal accuracy. Therefore, the heterogeneous surfaces of two microcantilevers remained unexpected in other chemical solutions.

### 3.2. Thermal Effect on Conventional Dual Free-Standing Microcantilevers

The response of a piezoresistive microcantilever is extremely sensitive to temperature change. Temperature changes in the surrounding environment may result in unwanted deflection and thus noise signals on the piezoresistive microcantilever biosensor. In this study, the thermal effects originated from the temperature coefficient of the resistor (TCR) and the inherently heterogeneous multilayer mismatch of thermal expansions. The resistance change was primarily attributed to the effect of the temperature coefficient of resistance. Furthermore, the microcantilever sensor was able to bend in response to temperature change due to the inherently heterogeneous multilayer mismatch of thermal expansions. The microcantilever structure consisted of multiple layers of Si_3_N_4_, poly-Si, SiO_2_ and gold, with each layer having its own inherent thermal expansion coefficient. In this study, the piezoresistor self-heating effect can be neglected because a low input voltage of 0.34 V was used.

In the conventional arrangement of a dual piezoresistive microcantilever sensor, the reference microcantilever was initially used to eliminate or reduce the thermal disturbance of its surrounding environment. To eliminate the thermal interference of microcantilever sensors, the symmetrical Wheatstone bridge electrical circuit configuration is commonly employed, which in this study consists of the gold-coated sensing piezoresistive cantilever, the silicon nitride reference piezoresistive cantilever and two external fixed resistors. However, a minute difference in material properties between the reference and gold-coated sensing microcantilevers still existed, leading to an inconsistent output with small temperature changes. The change in resistance of a piezoresistive microcantilever may be described by the expression:

(3) $$\frac{\Delta R}{R_{0}}=(\alpha_{\text{TCR}}+\alpha_{\text{Bimorph}})\Delta T$$

where *R_0_* is the resistance at the reference temperature *T_0_*, *α_TCR_* is the temperature coefficient, and *α_Bimorph_* is the effective thermal expansion coefficient of the multiple layers in the cantilever, which is often seen in the bimorph effect of two heterogeneous material layers. The changes in resistance of piezoresistors embedded in the reference and gold-coated microcantilever were measured as a function of temperature. The microcantilever sensor was placed on a Peltier element and heated from 20 °C to 45 °C. In order to avoid the self-heating effect of the piezoresistors, a bridge input voltage of 0.34 V was used. It was found that the effective (Δ*R*/*R*_0_)/Δ*T* value was 4.516 × 10^−4^ K^−1^ for the gold-coated microcantilever, and 4.509 × 10^−4^ K^−1^ for the reference microcantilever, respectively. As a result, both independently deflected cantilevers responded inconsistently, and exhibited a minute difference in resistance due to their material properties. Figure 2c shows that the voltage difference of 20~30 μV obtained from two terminals of the Wheatstone bridge demonstrated independent signals of both reference and sensing cantilevers with respect to the temperature variations. Figure 2d illustrates the result obtained by subtracting the reference beam deflection from that in the sensing one. As the temperature continued to increase, it was unpredictable whether the result would be positive or negative.

In a conventional dual microcantilever configuration, an additional gold layer on a sensing beam is required for sensing surface treatment, which might induce minute differences of surface curvature and thermal properties. The result indicated that two microcantilevers responded with nearly harmonious deflections to a stepped temperature increase, though with slight inconsistency. Ideally, the difference was expected to be constant and consistent.

In order to avoid unwanted disturbances caused by the reference microcantilever responding to changes in chemical substances and temperature in the liquid environment, a single free-standing sensing microcantilever was employed in this study to improve reproducibility and accuracy. However, the thermal issue remained a challenge for a single free-standing sensing microcantilever, which was very sensitive to temperature change, but had no reference cantilever to eliminate thermal disturbances. As a result, a reliable, stable temperature-controlled system was used in order to eliminate thermal interference from the environment or the device itself. This single microcantilever and thermally stable system design has long been used in cantilever measurement by optics-level technique. In this temperature-controlled system, the temperature variation can be controlled to within 0.1 °C, which effectively reduced the thermal interference on the piezoresistive microcantilever. Detection using a single free-standing microcantilever with a temperature-controlled system was made in the following experiment.

### 3.3. Detection of C-Reactive Protein

C-reactive protein an acute phase protein, and CRP levels during inflammation can increase by up to 100 or 500 times. The measurement of CRP levels in the blood has been suggested as an additional way to assess the risk of cardiovascular disease. A more sensitive CRP test, called a highly sensitive C-reactive protein (hs-CRP) assay, is required for the risk assessment of coronary heart disease (CHD) [29]. To serve as a biosensor, the physical sensing surface of a microcantilever was required to experience several major steps involving chemical and biochemical processes on its solid surface.

Meanwhile, the selected concentration of capture antibodies for immobilization was taken into account in this study, which may affect the electrostatic adsorption in the immobilization process. It has been noted in reference to commercial surface plasmon resonance biosensors [30] that a suitable concentration for the immobilization is suggested to be within the range between 10 μg/mL and 200 μg/mL. For a given pH, the ideal capture antibody concentration is the lowest value that results in the maximum immobilization density. Lower concentrations can impair the reproducibility of the immobilization, while the use of higher concentrations simply wastes capture proteins.

**Figure 3.**
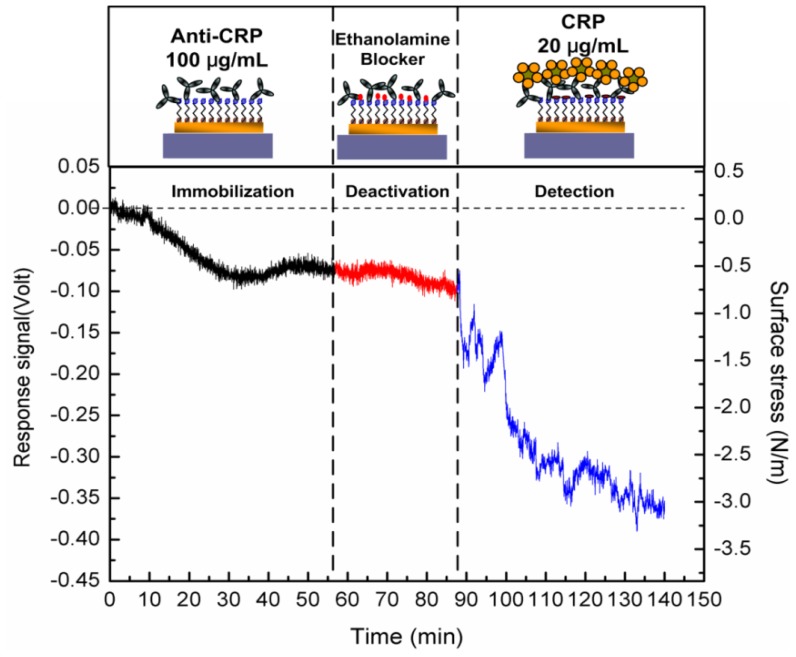
A continuous CRP detection signal (amplified signal) using a single free-standing temperature-controlled microcantilever biosensor responding to capture anti-CRP immobilization, deactivation and specific CRP binding interactions.

Figure 3 shows the complete curve of the microcantilever deflection in response to three major steps of the chemical and biochemical processes. The response of the microcantilever can be obtained in deflections or induced surface stresses. As demonstrated in Figure 3, the first stage was the response signal of the 100 μg/mL anti-CRP immobilization. The capture antibodies were covalently and randomly bonded onto the SAM layer. The induced change in surface stress was measured as 0.65 N/m, which led to a bend-down deflection (a compressive stress). An antibody conformation and a molecular-level force re-arrangement over 50 min achieved the required balancing force on the microcantilever. After inactivation or blocking of the functional bio-linker surfaces, no significant response was found. In other words, the sensing surface was mostly filled with immobilized capture antibodies in a 100 μg/mL concentration. After this, the immobilized antibody molecules were only exposed on the sensing surface for interaction with target antigens. As a result, the microcantilever was modified to be capable of detecting CRP antigens for specific recognition.

In the final step, a signal was generated by the specificity and the interaction of the antigen-antibody complexes. It took approximately 50 min to achieve an equilibrium state of the steady-state signal. The induced surface stress in the equilibrium state was 2.25 N/m for 20 μg/mL CRP detection. The continual deflection changes prior to the equilibrium state could result from a gradual rearrangement of the antibody-antigen complexes and conformational changes [31].

**Figure 4.**
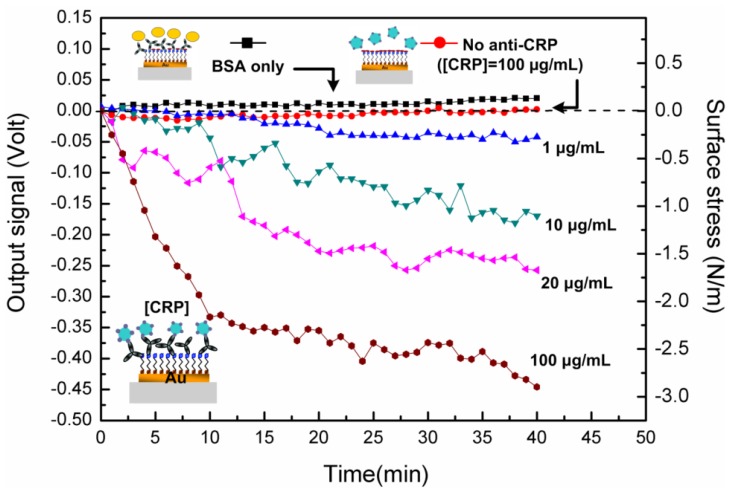
The electrical detection of various CRP concentrations between 1 μg/mL and 100 μg/mL was performed as a function of time using the single free-standing, thermally stable microcantilever biosensor. Specificity and no false positives were confirmed by negligible deflections of the non-immobilized microcantilever with an injection of 100 μg/mL CRP, and of the immobilized microcantilever with the injection of bovine serum albumin (BSA) in a high 1,000 μg/mL concentration. The responses were the amplified signals.

Figure 4 shows the piezoresistive response curves of the microcantilever with respect to various CRP concentrations between 1 μg/mL and 200 μg/mL, which is the clinically relevant range. The steady-state output signal was found to be correlated with the CRP concentration in solution. To verify the specificity of the detection, the microcantilever without anti-CRP on the SAM layer was exposed to a 100 μg/mL CRP solution, and the output signal was found to be negligible, as depicted in Figure 4. On the other hand, when the microcantilever immobilized with anti-CRP was exposed to incoming bovine serum albumin (BSA) at a high concentration of 1 mg/mL, no significant microcantilever deflection signal was observed.

**Figure 5.**
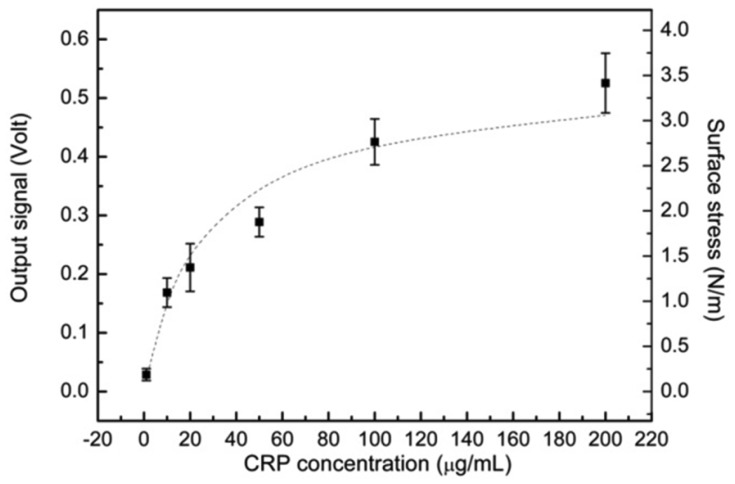
Immunoassay reproducibility and binding affinity investigation with respect to CRP concentrations measured by the electrical detection with single free-standing, thermally stable piezoresistive microcantilever biosensors.

In order to examine the reproducibility of the signal, we performed a series of experiments. Figure 5 shows the final steady output signals as a function of various analyte human CRP concentrations. The plot shows the reproducibility of the detection, which has a replication standard deviation (SD) of 0.039 Volt (∼0.25 N/m), and the coefficient of variation (CV) ranges from 17% to 21%. With a biosensing surface coverage of 100 μg/mL anti-CRP, the plot can be categorized into linear and saturated regions. In an approximately linear region, it was covered from 10 to 110 μg/mL; beyond this region, the signal was nearly saturated because most anti-CRP capture antibodies had been paired with CRP antigens. Only the remaining small binding sites were available for specific analyte detection.

Based on the results in Figure 5, the microcantilever measurement can be further studied to gain an insight into the biomolecular binding that can further ensure the valuable detection of this technique. The kinetics of biomolecular binding on microcantilever surfaces can be described using a simple Langmuir first-order scheme. The model assumes that the molecules are adsorbed at a fixed number of well-defined sites, each of which is energetically equivalent (a random process) and occupied by complete monolayer coverage when the surface reaches the saturation level. It is assumed that the rate constant for binding kinetics (association) is given by k_a_, whereas the rate constant for unbinding kinetics (dissociation) is given by k_d_. The rate of pair binding between the capture antibodies and antigens was given due to the random conjugation by [32]:

(4) $$\frac{dN}{dt}=k_{a}\Phi (N)C_{s}-k_{d}N$$

where N is the number of binding pairs on microcantilever surface as a function of time, C_s_ is the CRP concentration on the sensing surface, Φ(*N*) gives the remaining pairs available for binding. For a small ratio of k_d_/k_a_ with high affinity antibody–antigen interaction, the second term in the right hand side indicating dissociation process can be neglected [33]. We assumed that the response of the microcantilever from the conjugation pairs can be effectively contributed to the generation of surface stress. In practice, the correlation can be described in an equilibrium state by an input of dN/dt = 0, and in terms of the saturation level in equilibrium state to derive another form of kinetic basis systems [31]:

(5) $$\sigma_{eq.}=\frac{\sigma_{\max}C_{S}}{K_{D}+C_{S}}$$

since σ_eq_ and σ_max_ represent the equilibrium (steady state) and maximum values of surface stress, respectively; K_D_ is equal to k_d_/k_a_ and stands for the equilibrium dissociation constant or binding affinity. Asσ_eq_ is set to be atσ_max_/2, K_D_ can be determined and is equal to C_s_. In this study, the binding affinity (K_D_) was calculated to be 18.83 ± 2.99 μg/mL. Meanwhile, the capture protein in this study was used with mouse monoclonal anti-human CRP IgG1 molecules (Sigma C1688). As shown in reported results [34,35], the binding affinity of CRP-anti-CRP interaction was 3.53 × 10^−7^ M (41.7 μg/mL) in an evanescent wave field using microparticle-tracking velocimetry. The capture antibody was used with goat monoclonal anti-human CRP IgG molecules (Sigma C8284). Both CRP analytes were obtained in a purity of greater than 99%. The binding affinity obtained in this work was in the same order, and agreed with previous reported studies. Therefore, the reproducibility and CRP binding affinity proved to be valid detection measures using a single free-standing, thermally controlled piezoresistive microcantilever.

## 4. Conclusions

Electrical detection of CRP was successfully achieved using a single free-standing temperature-controlled piezoresistive microcantilever biosensor based on the Wheatstone bridge electrical circuit configuration. The piezoresistive microcantilever sensor was fabricated by the MEMS technique, and was highly compatible with the commercial semiconductor processes. Various CRP concentrations from 1 to 200 μg/mL, covering the clinically relevant range, were measured by the microcantilever biosensor as a function of time. The reproducibility and CRP binding affinity thus proved to be valuable tools in the context of cardiovascular disease risk assay and inflammation detection using the single free-standing, thermally controlled piezoresistive microcantilever biosensors.
